# Risk factors and outcomes associated with a higher use of inotropes in kidney transplant recipients

**DOI:** 10.1097/MD.0000000000005820

**Published:** 2017-01-10

**Authors:** Jae Moon Choi, Jun-Young Jo, Jae-Won Baik, Sooyoung Kim, Chan Sik Kim, Sung-Moon Jeong

**Affiliations:** Department of Anesthesiology and Pain Medicine, Asan Medical Center, University of Ulsan College of Medicine, Seoul, Republic of Korea.

**Keywords:** a higher use of inotropes, kidney transplant recipients, risk factors and outcomes

## Abstract

Preservation of adequate perfusion pressures to the graft is a main focus of intraoperative management during kidney transplantation. We undertook this study to investigate the incidence of the higher use of inotropes in kidney transplant recipients and identify the patient outcomes and preoperative and intraoperative variables related to this.

We retrospectively analyzed 1053 patients who underwent kidney transplantation at Asan Medical Center between January 2006 and February 2012, stratified by their inotropic score ([dopamine] + [dobutamine] + [epinephrine × 100] + [norepinephrine × 100]) <7 versus ≥7, wherein all doses are expressed as μg/kg/min. We evaluated preoperative characteristics, hemodynamic parameters, and intraoperative variables as well as postoperative outcomes, such as length of hospital stay and 1-year rejection and mortality rate.

Receiver-operating characteristic analysis was performed to determine inotropic score to predict 1-year mortality. An inotropic score of 7 had the best combined sensitivity and specificity. An inotropic score ≥7 (137 patients, 13.0%) was significantly more prevalent in older patients, those with polycystic kidney disease, and at a 2nd transplant. Anesthesia time, the amounts of crystalloid and 5% albumin infused, and the need for red blood cell transfusion were significantly higher in the inotropic score ≥7 group. The patients with a higher use of inotropes required longer postoperative hospital stay and experienced a >2-fold higher rejection within the 1st year and a 4-fold higher 1-year mortality rate.

A higher use of inotropes in kidney transplant recipients is more prevalent in older patients, those with a 2nd transplant and in patients with polycystic kidney disease as their primary renal disease. The postoperative hospital stay, rejection within the 1st year, and 1-year mortality rate are increased in patients with an inotropic score ≥7.

## 1 Introduction

Significant changes in blood pressure are common throughout the surgical procedure for kidney transplantation. The kidney transplantation procedure contains prolonged episodes of minimal stimulation. Maintenance of acceptable anesthetic depth to avoid awareness may reduce blood pressure and perfusion pressure to the newly reperfused kidney. In a large series of renal transplantations studied by Heino et al,^[1]^ hypotension (49.6%) was a more common finding than hypertension (26.8%). Hypotension is commonly encountered, especially after the fascia is dissected and might be further exacerbated after reperfusion of the graft.^[2]^

Allograft function, as determined by intraoperative urine production, is typically optimized by maintaining satisfactory intraoperative perfusion pressure.^[3]^ Preservation of adequate perfusion pressures to the graft is a main focus of intraoperative management during kidney transplantation. All efforts are made to maintain an appropriate level of blood pressure by using an adequate intravascular volume and vasopressor administration.^[4]^ Aggressive administration of fluid to optimize graft perfusion may be problematic in patients with a history of congestive heart failure and a low ejection fraction.^[2]^ Recipients undergoing kidney transplantation may suffer difficulty in maintaining adequate blood pressure without the use of inotropes. However, to our knowledge, there have been no previous studies assessing the requirements for vasoactive inotropes in patients undergoing kidney transplantation.

We undertook this study to investigate the incidence of a higher use of inotropes in kidney transplant recipients and identify preoperative and intraoperative variables related to this. In addition, we evaluated the association between the higher use of inotropes and patient outcomes.

## 2 Methods

After obtaining institutional review board approval, the electronic medical records for patients aged ≥18 years who underwent kidney transplantation at Asan Medical Center between January, 2006 and February, 2012 were retrospectively reviewed. Each transplantation procedure was evaluated and approved by the local authorities and the Korean Network for Organ Sharing affiliated with the Korean Ministry of Health and Welfare.

Kidney transplantation procedures were performed by a dedicated team using the standard anesthesia protocol at Asan Medical Center.^[5]^ After applying routine hemodynamic monitoring, general anesthesia was induced with 5 mg/kg thiopental, 50 to 100 μg fentanyl, and 0.5 mg/kg atracurium. Following endotracheal intubation, anesthesia was maintained using isoflurane or desflurane, a 50% O_2_/nitrous oxide mixture, and atracurium. Mechanical ventilation was performed using a constant tidal volume of 8 to 10 mL/kg and a respiratory rate of 10 to 12 cycles/min to maintain a constant end-tidal CO_2_ partial pressure of 30 to 35 mm Hg. Fluid management with crystalloid and albumin solution was performed based on a target central venous pressure level of 12 mm Hg, and packed red blood cells were transfused if the hematocrit was <21%.

The variables analyzed for patient characteristics included age and gender, history of renal transplant, dialysis technique, duration of dialysis, preoperative systolic blood pressure (SBP), heart rate, hemoglobin, and creatinine value. Their primary renal disease and type of renal transplant were also reviewed. Intraoperative variables regarding the anesthesia time, crystalloid infused, 5% albumin infused, transfusion of red blood cell, and urine output were obtained. Postoperative variables included the length of postoperative hospital stay, rejection within the 1st year following transplantation, and 1-year mortality.

In some patients in which an SBP <120 mm Hg persisted after repeated fluid boluses, vasoactive-inotropic drugs were infused appropriately for hemodynamic stability. To quantify the intraoperative requirement for vasoactive-inotropic drugs, we chose to use the maximum inotropic score for each patient. The inotropic score was obtained as follows: [dopamine dose] + [dobutamine dose] + [epinephrine dose × 100] + [norepinephrine dose × 100], wherein all doses are expressed as μg/kg/min.^[6,7]^

Continuous data were tested for a normal distribution using the Shapiro–Wilk test. Data were presented as a mean ± SD for normally distributed continuous variables and a number (%) for categorical variables. Receiver operating characteristic analysis was used to determine the optimal cut-off value of inotropic score. A student *t* test was used to compare the means of quantitative continuous variables. Categorical data were compared with a chi-square test or Fisher exact test to appropriately assess differences between the 2 groups.

Cumulative survival rates were calculated using the Kaplan–Meier method, and differences between curves were evaluated using the log-rank test. Statistical analyses were performed using SPSS version 21.0 (IBM Corp., Armonk, NY) with a significance level set at 5%.

## 3 Results

A total of 1060 renal transplant recipients were initially evaluated of which 7 patients with incomplete data were excluded from the subsequent analysis. Ultimately, 1053 renal transplant recipients were included in this study. A receiver operating characteristic analysis was performed to determine an inotropic score to predict 1-year mortality. An inotropic score of 7 had the best combined sensitivity (39.9%) and specificity (87.7%), and the area under the curve was 0.631 (95% CI 0.601–0.660, *P* = 0.017). Of these, the number of patients with an inotropic score ≥7 was 137 (13.0%).

The patient preoperative characteristics, primary renal disease, and type of renal transplant are listed in Table 1. A total of 905 patients received living donor transplants (85.9%), and 148 received organs from deceased donors (14.1%). When we compared the recipient profiles between groups with an inotropic score <7 versus ≥7, most preoperative characteristics did not show statistically significant differences. An inotropic score ≥7 was significantly more prevalent in older patients and in recipients of 2nd transplant, in patients with polycystic kidney disease, and a deceased donor kidney transplant. The dialysis technique and duration of dialysis did not differ between the 2 groups.

**Table 1:**
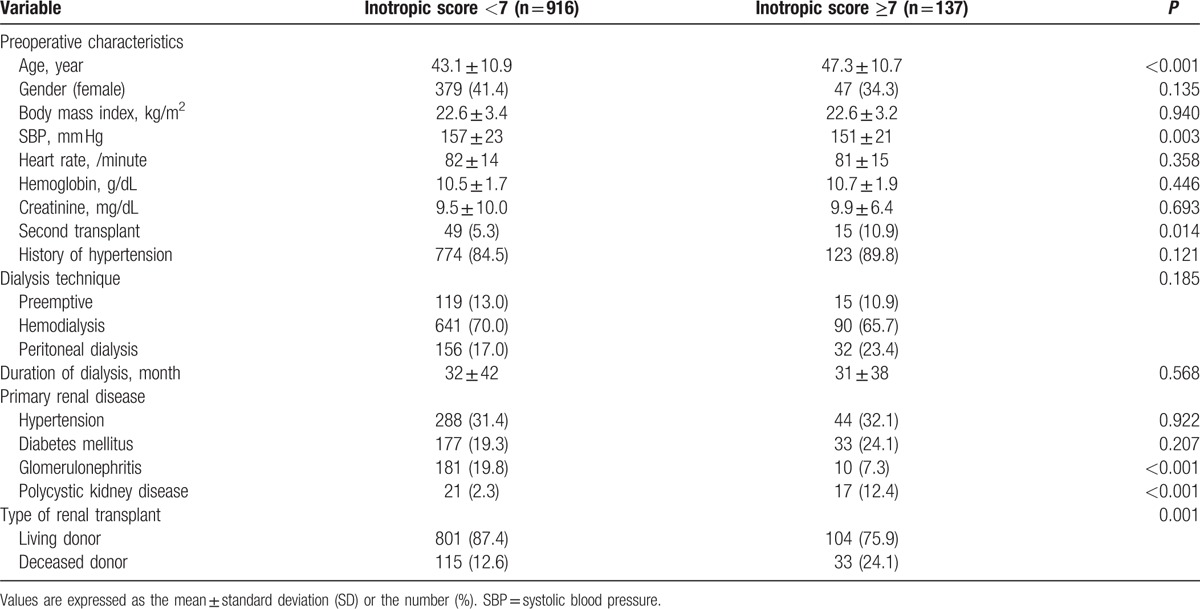
Preoperative variables, dialysis techniques, primary renal disease, and the type of renal transplant in patients with inotropic score <7 versus ≥7.

**Figure 1:**
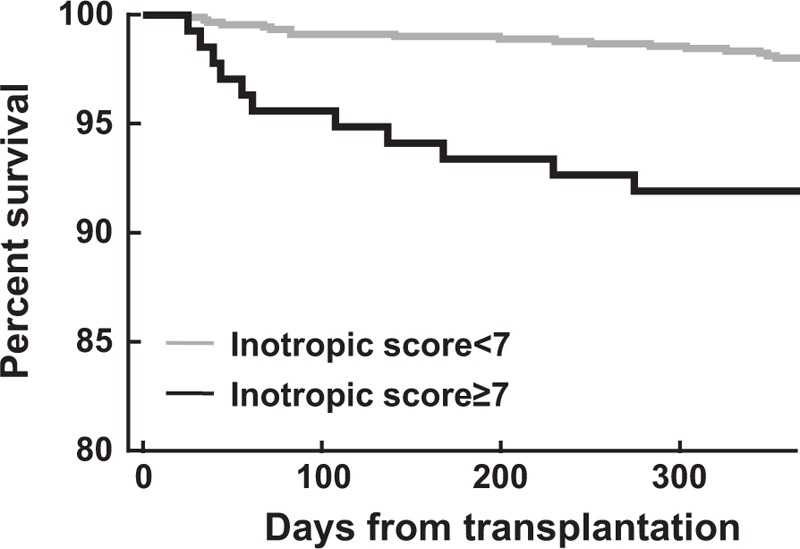
Kaplan–Meier unadjusted patient survival for patients who had an inotropic score ≥7 (black line) compared with those who had an inotropic score <7 (gray line). Survival differences between the groups was highly significant (log-rank test, *P* < 0.001).

Anesthesia time, the amounts of crystalloid and 5% albumin infused, and the need for red blood cell transfusion were significantly higher in the group with an inotropic score ≥7. The required postoperative hospital stay was 16.1 ± 8.6 days in the group with an inotropic score <7 versus 19.0 ± 10.9 days in the group with an inotropic score ≥7 (*P* < 0.001). Thirteen (9.5%) of an inotropic score ≥7 had an episode of rejection within the 1st year following transplantation compared with 3.7% of an inotropic score <7 (*P* = 0.006). The 1-year mortality was also increased in patients with an inotropic score ≥7 compared with those with an inotropic score <7 (8.0% vs 2.0%, *P* = 0.001; Table 2). Figure 1 shows the survival analysis with a Kaplan–Meier curve indicating that the group with an inotropic score ≥7 displayed increased overall mortality (log-rank test, *P* *<* 0.001).

**Table 2:**
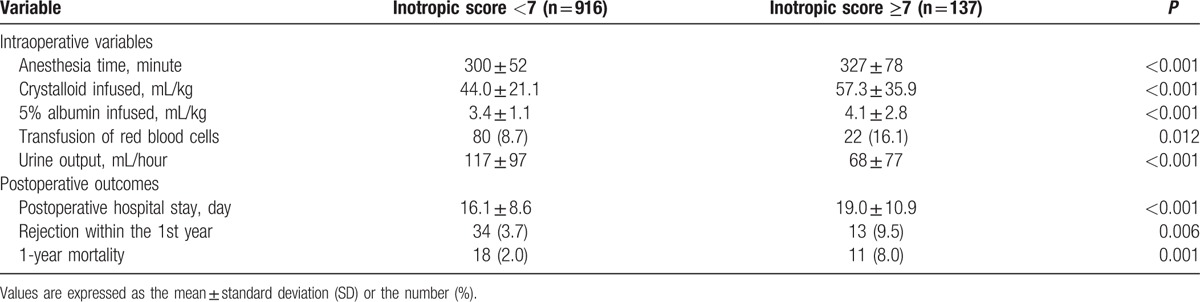
Intraoperative variables and postoperative outcomes in patients with an inotropic score <7 versus ≥7.

## 4 Discussion

In the present study, we investigated the preoperative characteristics, intraoperative variables, and postoperative outcomes between renal transplant recipients with an inotropic score <7 versus an inotropic score ≥7 in a cohort of 1053 patients. Older patients, patients undergoing 2nd transplant, and those with polycystic kidney disease as their primary renal disease more often showed an inotropic score ≥7. Anesthesia time, the amounts of crystalloid and 5% albumin infused, and the need for red blood cell transfusion were significantly higher in the inotropic score ≥7 group. The patients with a higher use of inotropes required longer postoperative hospital stay and experienced a >2-fold higher rejection within the 1st year and a 4-fold higher 1-year mortality rate.

It is essential to avoid hypotensive episodes after reperfusion. Several studies advise that anesthesiologists should maintain the recipient's SBP above 120 mm Hg and mean arterial pressure over 95 mm Hg during kidney transplantation.^[8,9]^ These values are intended to ensure sufficient perfusion pressure of the graft and rapid recovery of renal function. Improved oxygenation of the graft immediately after reperfusion results in a decreased incidence of delayed graft function.^[10]^ Hypotension will result in hypoperfusion of the graft and eventually can delay the return of function and precipitate renal injury. Adequate intravascular volume expansion with crystalloids, colloids, or both is important to increase renal blood flow, which improves immediate graft function in a renal transplant.^[11]^ Mannitol or loop diuretics are also administered before reperfusion to stimulate diuresis.

Hypotension unresponsive to volume expansion may require administration of vasoactive inotropes. In our study, the proportion of patients with an inotropic score ≥7 was 13.0%. Potura et al^[12]^ reported that 22.6% of patients with end-stage renal disease undergoing a deceased renal transplant required catecholamines to maintain a mean arterial pressure above 60 mm Hg. Although a clear recommendation for the use of vasoactive-inotropic drugs during kidney transplantation cannot be made,^[13]^ we used vasopressors to maintain an SBP >120 mm Hg.

In our present study, the postoperative hospital stay duration, rejection within the 1st year, and 1-year mortality rate were significantly increased in patients with an inotropic score ≥7. Low-dose dopamine may improve transplanted kidney function with consequent increases in effective renal plasma flow.^[14]^ However, it has been found in rats that kidney transplantation changes resistance vessels, causing them to respond more intensely to constrictor stimuli.^[15]^ The responses to sympathomimetics in grafted rat kidney were also found to shift towards a reduction in renal blood flow.^[16]^ In addition, the use of dopamine during kidney transplantation had no beneficial effect on early graft function.^[17]^ Ciapetti et al^[18]^ also reported a higher mortality and prolonged intensive care unit stays in patients receiving dopamine after renal transplantation. Cardiovascular morbidity was increased through alterations of arterial function characterized by diminished distensibility of large arteries.^[19]^ These correlations might be explained by increased cardiovascular morbidity in renal transplant recipients receiving inotropes.

Our present study had several limitations. Our study population was derived from a single large center. Local perioperative management strategies for kidney transplantation may have influenced the use of vasoactive-inotropic drugs, perioperative variables, and the outcome. Another limitation of our study was that invasive arterial blood pressure monitoring has been used as a guideline for the use of inotropic drugs without the use of cardiac output monitoring. An additional possible limitation was due to the study design as a retrospective observation analysis; therefore, careful interpretation is required to evaluate the potential association between an inotropic score ≥7 and the outcome. Moreover, it has not yet been determined what kind of blood pressure management is suitable in renal transplant recipients who do not respond to fluid therapy. For patients who did not respond to fluid infusion, further studies are needed to compare the clinical outcomes in patients who used inotropes to ensure sufficient graft perfusion pressure to those who did not use inotropes, thus allowing lower blood pressure in these patients.

In conclusion, a higher use of inotropes in kidney transplant recipients is more prevalent in patients with increased age, with 2nd transplant, and with polycystic kidney disease as the primary renal disease. The postoperative hospital stay, rejection within the 1st year, and 1-year mortality rate are increased in patients with an inotropic score ≥7.

## Acknowledgments

The authors thank Seunghee Baek, PhD (Department of Clinical Epidemiology and Biostatistics, Asan Medical Center, Seoul, Korea) for statistical consultation.
